# Identification of Hypoxia–Immune-Related Gene Signatures and Construction of a Prognostic Model in Kidney Renal Clear Cell Carcinoma

**DOI:** 10.3389/fcell.2021.796156

**Published:** 2022-02-08

**Authors:** Shuheng Bai, Ling Chen, Yanli Yan, Xuan Wang, Aimin Jiang, Rong Li, Haojing Kang, Zhaode Feng, Guangzu Li, Wen Ma, Jiangzhou Zhang, Juan Ren

**Affiliations:** ^1^ Department of Radiotherapy, Oncology Department, First Affiliated Hospital of Xi’an Jiaotong University, Xi’an, China; ^2^ Department of Chemotherapy, Oncology Department, First Affiliated Hospital of Xi’an Jiaotong University, Xi’an, China; ^3^ Medical School, Xi’an Jiaotong University Xi’an, Xi’an, China

**Keywords:** hypoxia, kidney clear cell carcinoma, prognosis, immune infiltrate cells, bioinformatic analysis

## Abstract

**Introduction:** Kidney renal clear cell carcinoma (KIRC), a kind of malignant disease, is a severe threat to public health. Tracking the information of tumor progression and conducting a related dynamic prognosis model are necessary for KIRC. It is crucial to identify hypoxia–immune-related genes and construct a prognostic model due to immune interaction and the influence of hypoxia in the prognosis of patients with KIRC.

**Methods:** The hypoxia and immune status of KIRC patients were identified by utilizing t-SNE and ImmuCellAI for gene expression data. COX and Lasso regression were used to identify some hypoxia–immune-related signature genes and further construct a prognostic risk model based on these genes. Internal and external validations were also conducted to construct a prognostic model. Finally, some potentially effective drugs were screened by the CMap dataset.

**Results:** We found that high-hypoxia and low-immune status tend to induce poor overall survival (OS). Six genes, including PLAUR, UCN, PABPC1L, SLC16A12, NFE2L3, and KCNAB1, were identified and involved in our hypoxia–immune-related prognostic risk model. Internal verification showed that the area under the curve (AUC) for the constructed models for 1-, 3-, 4-, and 5-year OS were 0.768, 0.754, 0.775, and 0.792, respectively. For the external verification, the AUC for 1-, 3-, 4-, and 5-year OS were 0.768, 0.739, 0.763, and 0.643 respectively. Furthermore, the decision curve analysis findings demonstrated excellent clinical effectiveness. Finally, we found that four drugs (including vorinostat, fludroxycortide, oxolinic acid, and flutamide) might be effective and efficient in alleviating or reversing the status of severe hypoxia and poor infiltration of immune cells.

**Conclusion:** Our constructed prognostic model, based on hypoxia–immune-related genes, has excellent effectiveness and clinical application value. Moreover, some small-molecule drugs are screened to alleviate severe hypoxia and poor infiltration of immune cells.

## Introduction

Cancer is a major public health problem worldwide and is the second leading cause of death in the United States (Siegel et al., 2018). In 2020, about 73,750 newly diagnosed renal cancer cases and 14,830 deaths were registered. The incidence rates in male and female are 5 and 3%, respectively (Siegel et al., 2020). Kidney renal clear cell carcinoma (KIRC) is the most representative subtype of renal cancer, accounting for about 75% of all patients with renal cancer, with an increased incidence rate year by year (Linehan and Ricketts, 2019; Bai et al., 2020a). However, KIRC-related clinical symptoms and biomarkers are lacking. Hence, KIRC cannot be diagnosed early, resulting in poor response to conventional therapy and poor survival rate (Bai et al., 2020b). Thus, KIRC is a threat to public health as a malignant disease, and it is necessary to conduct real-time information tracking and dynamic prognosis analysis for KIRC patients (Zhao et al., 2018). TNM staging is a classical manner to predict cancer prognosis and suggest treatment strategies. However, the classification of TNM is based on clinical information, and it does not consider any genetic features (Choi et al., 2017; Wang et al., 2021a). So, it is of great significance and urgency to identify some novel effective gene signatures for KIRC.

Recently, immune therapy has been proven highly efficacious in KIRC; especially immune checkpoint inhibitors that block PD-1/PD-L1 or CTLA-4 T cell inhibitory receptors are considered standard treatment (Motzer et al., 2015; Motzer et al., 2018). KIRC is heterogeneous, such that the tumor cells are in an intricate tumor microenvironment, including immune cells, spongiocytes, fibroblasts, and vessels. Features of the infiltration immune cells in the tumor microenvironment can heavily affect the responses to systemic therapy (Vuong et al., 2019). Hypoxia is a typical hallmark in nearly all solid tumors, arising from the rapid and uncontrolled proliferation of tumors and insufficient blood supply, which plays a vital role in tumor genetic instability and prognosis (Schödel et al., 2016; Shao et al., 2018). Hypoxia also plays a critical role in cell proliferation, differentiation, apoptosis, and tumor angiogenesis (Haase, 2006; Gonzalez et al., 2018; Zhang et al., 2020a). Some studies have shown that hypoxia can regulate the status of the tumor immune microenvironment, such as promoting the recruitment of innate immune cells and interfering with the differentiation and function of adaptive immune cells, which finally leads to the consequent immunosuppression and immune evasion of the tumor (Palazon et al., 2014; Terry et al., 2017; Jing et al., 2019; Wang et al., 2021b). HIFs are dimeric proteins consisting of an O_2_-sensitive a subunit (HIF-1a, HIF-2a, or HIF-3a) and a scaffold b subunit (HIF-2b) that play an important role in mediating hypoxia-related biological processes (Shi et al., 2021; You et al., 2021). Under hypoxia conditions, HIFs can bind with transcriptional coactivator and hypoxia response element to increase the expression of a string of target genes, consequently regulating various biological processes, including proliferation, metabolism, angiogenesis, migration, and invasion (Gilkes et al., 2014; de Heer et al., 2020; Hoefflin et al., 2020; Yang et al., 2020). Therefore, immune-hypoxia-related genes may be effective signatures for predicting the overall survival (OS) outcomes of KIRC patients.

In this research, we hypothesized that immune and hypoxia interaction might greatly influence the prognosis of patients with KIRC by identifying a string of hypoxia–immune-related genes. On that basis, we constructed a novel prognostic risk model and screened some potentially effective drugs to improve the KIRC prediction (d≤iagnosis) and understand its underlying mechanism.

## Methods

### Data Acquisition

All data about kidney renal clear cell carcinoma in this study were obtained from The Cancer Genome Atlas (TCGA) and Gene Expression Omnibus (GEO). The TCGA cohort total included 511 records of patients with KIRC, whose profiling data (level 3, “”FPKM workflow) and corresponding clinical data were obtained from the TCGA official website (https://cancergenome.nih.gov). Furthermore, the gene expression profile of 72 normal samples (normal tissue adjacent to the tumor in the same patient) was also obtained from TCGA. The expression matrix and clinical information of GSE29609 were downloaded from GEO. It was based on the GPL1708 platform and contained 39 KIRC samples. Background correction and quality normalization were performed for GEO profiling data by applying the multiarray average algorithm.

In our research, the patients from TCGA-KIRC were defined as a training cohort, and the patients from GSE29609 were utilized as an external validation cohort. The detailed clinical information about the training cohort and the external validation cohort are shown in Table 1, including age, gender, tumor stage, and pathological grade.

**TABLE 1:** Detailed clinical information about the training cohort and external validation cohort.

Characteristics	Group	TCGA-KIRC (*N* = 511)		GEO-GSE29609 (*N* = 39)	
		Number	%	Number	%
Age	≤60	260	50.9	16	41.0
	＞60	251	49.1	23	59.0
Tumor stage	I	255	49.9	10	25.6
	II	53	10.4	3	7.7
	III	118	23.1	12	30.8
	IV	85	16.6	14	35.9
Pathological grade	G1	12	2.3	1	2.6
	G2	217	42.5	12	30.8
	G3	202	39.5	11	28.2
	G4	75	14.7	15	38.5
	Unknown	5	1.0	0	0.0
Vital status	Alive	346	67.7	23	59.0
	Dead	165	32.3	16	41.0

### Identification of Hypoxia Status

Algorithms of t-distributed Stochastic Neighbor Embedding (t-SNE) and K-Means Clustering (K-means) were utilized to deduce the hypoxia status of tumor samples, which can be applied from Rtsne and k-means R software packages. T-SNE is a nonparametric, unsupervised method that divides or condenses patients into several distinct clusters based on given signatures or hallmarks. This present research included 79 genes as adopted hallmarks of hypoxia genes, which were obtained by following these two procedures. Firstly, 200 genes were obtained from collecting hypoxia-related hallmark gene sets in the Molecular Signatures Database (MsigDB V7.4). Secondly, univariate Cox regression analysis was used to select from these genes, which was performed by Survival R-package, and finally, the hypoxia-related genes utilized in this research were obtained. Based on the algorithms mentioned above, the patients were divided into groups depending on hypoxia status. Expression changes analysis about HIF-1 pathway-related genes and survival analysis were conducted to explore the difference between hypoxia-low and hypoxia-high groups. The primary genes involved in the HIF-1 signaling pathway were extracted from the Kyoto Encyclopedia of Genes and Genomes (KEGG) database. Among the retrieved 26 genes, 16 were involved in the “increase oxygen delivery” cluster, and 11 were related to the “reduce oxygen consumption” cluster. In addition, HIF-1α was artificially added to these gene sets. The expression change analysis was conducted by the Limma package and EdgeR package of R software. Genes with a false discovery rate (FDR) adjusted *p*-value < 0.05 and an absolute value of log2 (fold change) >1 were considered to be of statistical significance.

### Identification of Immune Infiltration Status

ImmuCellAI (Immune Cell Abundance Identifier) is a newly developed web tool (http://bioinfo.life.hust.edu.cn/ImmuCellAI/), which aims to estimate the abundance of 24 immune cells from a gene expression dataset including RNA-Seq and microarray data. A major advantage of ImmuCellAI is providing an infiltration score to represent the overall infiltration level of immune cells for each cancer sample (Miao et al., 2020). So, in this research, ImmuCellAl was used to predict the immune status and provided the basis for dividing the samples into immune-high and immune-low groups. We identified the best optimal cutoff value by dividing the samples with the most significant outcomes. A function “surv_cutpoint” from the Survminer R software package was applied in our research to determine the optimal cutoff value for one or multiple continuous variables at once.

### Dividing Into Groups Based on Hypoxia–Immune Status

The identification of hypoxia and the immune status of each patient have been described above. All TCGA samples were labeled with two-dimension contributions and divided into three groups, including “hypoxia-high + immune-low group,” “hypoxia-low + immune-high,” and “mixed group,” which contained “hypoxia-high + immune-high group” and “hypoxia-low + immune-low group.” Survminer R package was utilized to carry out a survival analysis for these three groups. Moreover, the Limma package was used to obtain the preliminary hypoxia–immune-related differentially expressed genes (DEGs).

In the same way, the DEGs between tumor samples and normal samples were also achieved. Two sets of genes (hypoxia–immune-related protective and risk DEGs) were also developed by overlapping the hypoxia–immune-related DEGs and tumor–normal-related DEGs.

### KEGG Pathway, GO Enrichment Analysis, and Construction of the PPI Network

To understand the functions and pathways of these risk or protective DEGs obtained as detailed above, KEGG pathway analysis and GO enrichment analysis were conducted by applying clusterProfiler R package. Both *p*- and FDR values less than 0.05 were statistically significant.

Protein–protein interaction (PPI) networks were also constructed to further screen the key module for risk genes and protective genes. PPI networks of risk or protective genes were constructed using the Search Tool for the Retrieval of Interacting Genes database (STRING, version 11.0) to provide credible information in interactions between proteins and supply detailed annotation (Szklarczyk et al., 2019). Cytoscape (version 3.7) is a general-purpose, open-source software (Shannon et al., 2003), which was further employed to build PPI networks. The crucial modules were screened using the Molecular Complex Detection module with a criterion score ≥5.

### Building and Verifying the Hypoxia–Immune-Related Prediction Model

The risk and protective gene sets mentioned above were normalized by using log2 transformation. Univariate Cox and least absolute shrinkage and selection operator (LASSO) regression were conducted using the Survival and Glmnet R package. Afterward, multivariate Cox regression analyses were utilized to construct a hypoxia–immune-related prediction model. The risk score was calculated using the following formula: risk score = $\overset{n}{\underset{i=1}{\sum }}coef\times gene$ . Kaplan–Meier survival analysis was also carried out to assess the difference in survival between high- and low-risk-score groups by using the “survival” R package. GEPIA was utilized again to verify the influence on the expression and prognosis by these genes contained in the prediction model (Tang et al., 2017).

We verified the performance of the gene risk model by comparing the prediction efficiency with the clinical features. The time-dependent receiver operating characteristic (ROC) curve was used by applying the “survivalROC” R package. The decision curve analysis (DCA) algorithm from the ggDCA R package was also conducted to determine the clinical usefulness of the gene risk model by quantifying the net benefits at different threshold probabilities. In addition, the ROC and DCA analyses were also conducted for GSE29609, which were used as external validation.

### Construction and Validation of a Predictive Nomogram Consisting of Risk Score and Clinical Features

A nomogram was built using the rms R package, and it included the risk score calculated above and some clinical features, such as clinical stage, pathological grade, gender, and age. The calibration plot was applied to explore the calibration and discrimination of the nomogram by utilizing the rms R package.

### Identification of Candidate Small-Molecule Drugs Based on Hypoxia–Immune DEGs

The Connectivity Map (CMap) is a gene expression profiling database. It has excellent potential for discovering new therapeutic drugs for a disease, and it was used in this study to research on some small-molecule therapeutic drugs (Bai et al., 2020b; Lamb et al., 2006). By uploading the risk and protective gene sets respectively into the CMap website (https://portals.broadinstitute.org/cmap/), some candidate compounds were discovered to reverse the status of high-hypoxia and low-immune in KIRC patients. Negatively related drugs [*p* < 0.05, *n* ≥ 5, and mean score <−0.4 (Chen et al., 2020)] were screened, which had potential antagonistic effects, indicating that they could reverse the high-hypoxia and low-immune status and could serve as therapeutic drugs. Finally, the 3D structure data of these selected compounds were obtained from the ZINC dataset and represented by Pymol software (Sterling and Irwin, 2015).

## Results

### Identification of Hypoxia Status and Immune Status

**FIGURE 1:**
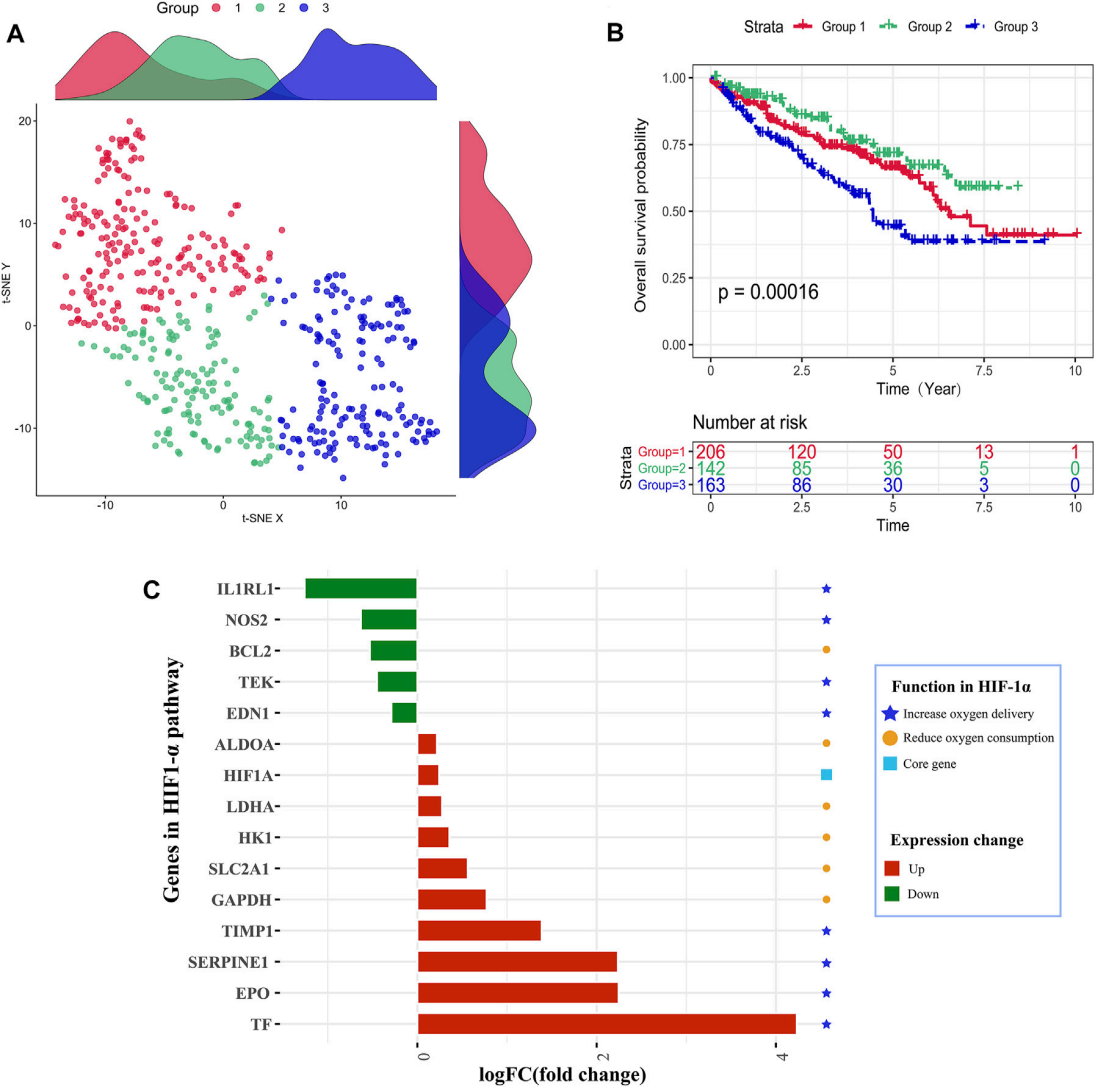
Identification of hypoxia status. **(A)** Dot plot for three distinct clusters identified by *t*-SNE and K-means algorithms based on 79 hypoxia-related genes. **(B)** Survival analysis (Kaplan–Meier) of overall survival for patients in three clusters. **(C)** Expression changes analysis about HIF-1 pathway-related genes to explore the difference between the hypoxia-high and hypoxia-low groups.

As shown in Figure 1A, the TCGA samples were divided into three groups based on hypoxia status by utilizing the t-SNE algorithm, and each group had 206, 142, and 163 samples, respectively. Kaplan–Meier survival analysis discovered that group 2 had the best overall survival, while group 3 had the worst prognosis. It indicated that groups 2 and 3 might be in the lowest and highest hypoxia status. So, we explore the gene expression changes of the HIF-1α signal pathway, which contains 29 genes and can be classified into three gene sets: (1) 15 genes about “increase oxygen delivery,” (2) 13 genes about “reduce oxygen consumption,” and (3) the core gene “HIF-1A.” As shown in Figure 1C, four genes (TIMP1, SERPINE1, EPO, and TF) had higher expression levels in the hypoxia-high group, and only one gene (IL1RL1) was overexpressed in the hypoxia-low group. We found that the expression changes of upregulated genes were higher than those of downregulated genes. These results showed that the defined groups were significantly associated with hypoxia. Meanwhile, patients who were classified in group 2 were labeled hypoxia-high and those in group 3 were assigned as hypoxia-low.

**FIGURE 2:**
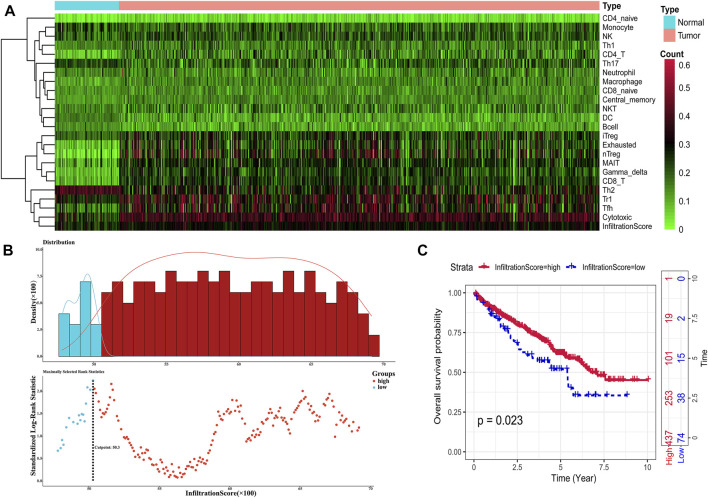
Identification of immune infiltration status. **(A)** Heat map showing the infiltration characteristics of all immune cells for all samples. **(B)** Scatter plot shows the optimal cutoff value for the immune scores of the tumor samples. **(C)**. Kaplan–Meier plots of overall survival for patients in the immune-high and immune-low groups.

ImmuCellAl calculated the infiltration proportions of all 24 immune cells of all TCGA samples, and we could find that the infiltration characteristics of all immune cells were obviously different between the tumor sample and the normal sample, especially in terms of Treg, CD8-T, cytotoxicity, infiltration score, and so on (Figure 2A). ImmuCellAl also provided an infiltration score term for all samples, which ranged from 0.351 to 0.9, representing the overall level of the infiltration proportion of all immune cells in patients. Based on the infiltration score, we identified the optimal cutoff value to classify patients into immune-high and immune-low groups with the most distinct prognosis by applying the “surv_cutpoint” function of the Survminer R package (Figure 2B). The result of the survival analysis demonstrated that a total of 437 patients in the immune-high group apparently represented better survival outcomes than those in the immune-low group (Figure 2C).

### Dividing Into Groups and Getting Risk and Protective DEGs Based on Hypoxia–Immune Status

**FIGURE 3:**
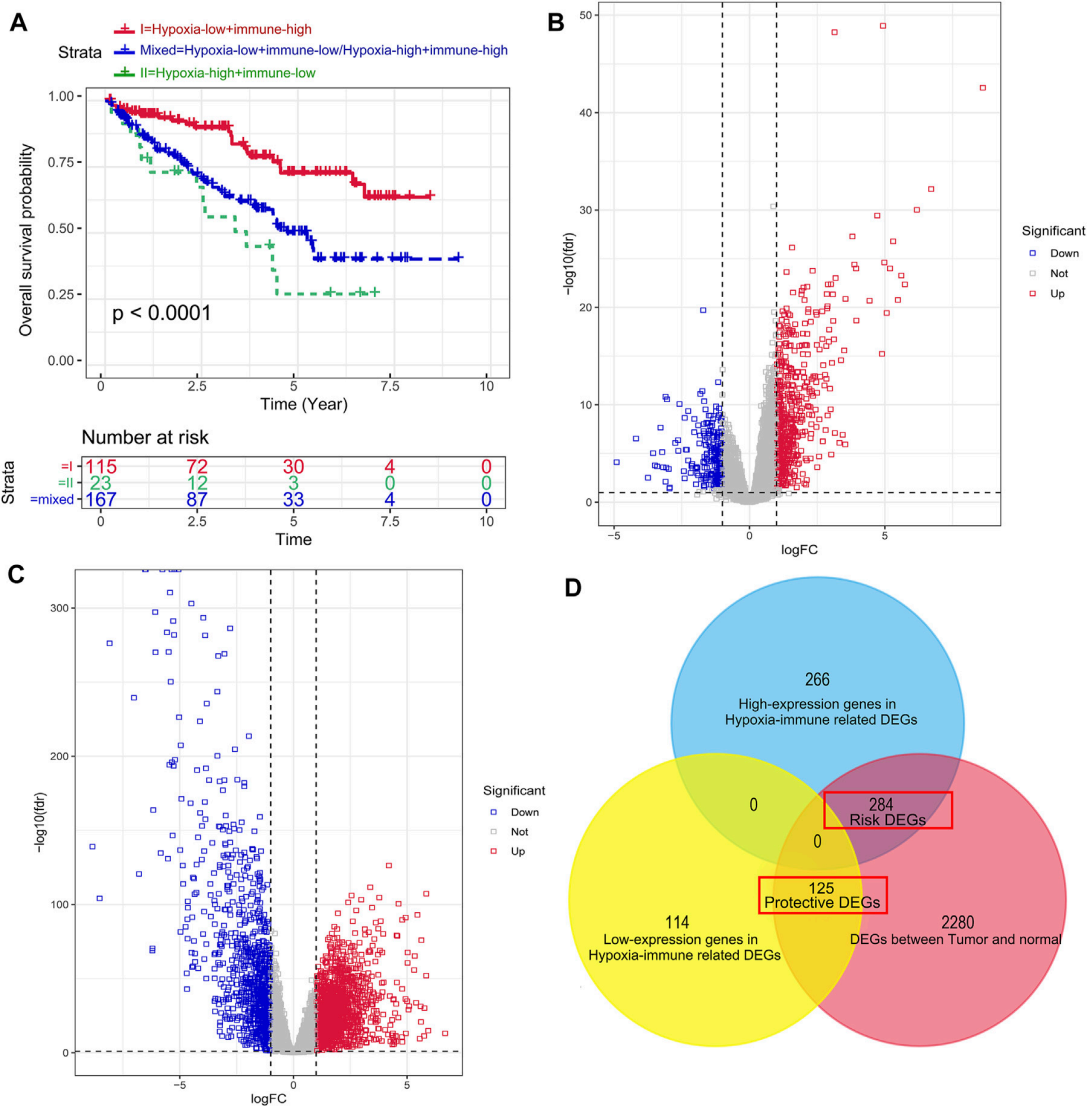
Dividing into groups and getting risk and protective differentially expressed genes (DEGs) based on hypoxia–immune status. **(A)** Kaplan–Meier plot of OS for patients in group I (hypoxia-low + immune-high), group II (hypoxia-high + immune-low group), and group mix (hypoxia-high + immune-high group and hypoxia-low + immune-low group). **(B)** Volcano plot showing the DEGs between group I and group II. **(C)** Volcano plot showing the DEGs between tumor samples and normal samples. **(D)** Venn diagrams showing overlaps of hypoxia–immune-related risk DEGs and hypoxia–immune-related protective DEGs.

According to the hypoxia and immune status identified above, we further combined them into a two-dimensional index, whereby TCGA patients could be divided into three groups: (1) group I was “hypoxia-low + immune-high,” (2) group II was “hypoxia-high + immune-low group,” and (3) group mix included “hypoxia-high + immune-high group” and “hypoxia-low + immune-low group.” The survival analysis showed a significant difference among these three groups, wherein patients in group I had a better prognosis. In contrast, the survival of patients in group 2 was worst, as shown in Figure 3A. Furthermore, this indicated that a more severe hypoxia status and a lower level of immune cell infiltration could induce a more severe prognosis in patients with KIRC.

To get the DEGs related to hypoxia and immune status, we conducted different expression change analyses between group 1 (hypoxia-low + immune-high) and group 2 (hypoxia-high + immune-low group), and a total of 789 DEGs were preliminarily obtained, as shown in Figure 3B. Then, in order to narrow down the scope of their DEGs, we achieved DEGs between tumor samples and normal samples (Figure 3C) and overlapped the hypoxia–immune-related DEGs and tumor–normal-related DEGs. Finally, 409 hypoxia–immune-related DEGs were identified, including 284 overexpressed genes in group 2, which were defined as hypoxia–immune-related risk DEGs, and 125 overexpressed genes in group 1, which were defined as hypoxia–immune-related protective DEGs (Figure 3D). It suggested that a higher expression of hypoxia–immune-related risk DEGs would lead to a poor prognosis; however, a higher expression of protective DEGs would cause a better prognosis.

### Enrichment Analysis for the Hypoxia–Immune-Related DEGs

**FIGURE 4:**
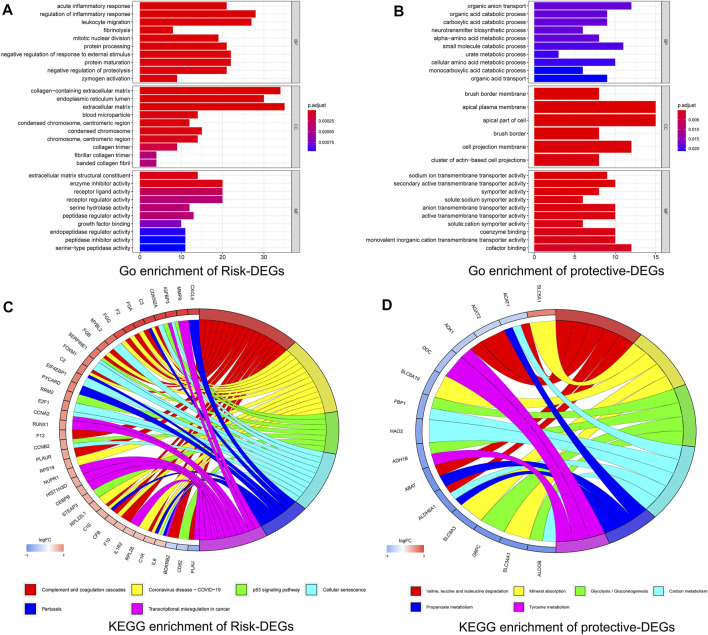
Enrichment analysis for the hypoxia–immune-related differentially expressed genes (DEGs). **(A**, **B)** Column diagrams of Gene Ontology analysis for hypoxia–immune-related DEGs. **(C**, **D)** Circos plots of Kyoto Encyclopedia of Genes and Genomes analysis for hypoxia–immune-related DEGs.

To further explore the hypoxia–immune-related DEGs obtained as detailed above, we conducted KEGG pathway enrichment analysis and GO function enrichment analysis. It can be seen from Figure 4A that the risk DEGs were mainly enriched in BP, including “acute inflammatory response,” “regulation of inflammatory response,” “leukocyte migration,” *etc*., which were primarily related to the function of the immune response. The BP analysis of protective DEGs primarily included “organic anion transport,” “organic acid catabolic process,” “carboxylic acid catabolic process,” *etc*., which were correlated with the regulation of surrounding acid (Figure 4B).

The KEGG pathway analysis showed that the risk DEGs were mainly enriched in some cancer or immune-related pathways, including “complement and coagulation cascades,” “p53 signaling pathway”, “transcriptional misregulation in cancer,” and so on (Figure 4C). The protective DEGs also mainly included “valine, leucine, and isoleucine degradation” and “mineral absorption” (Figure 4D). All these results indicated that these DEGs were closely correlated with immune and cancer pathways.

### PPI Network of Hypoxia–Immune DEGs

**FIGURE 5:**
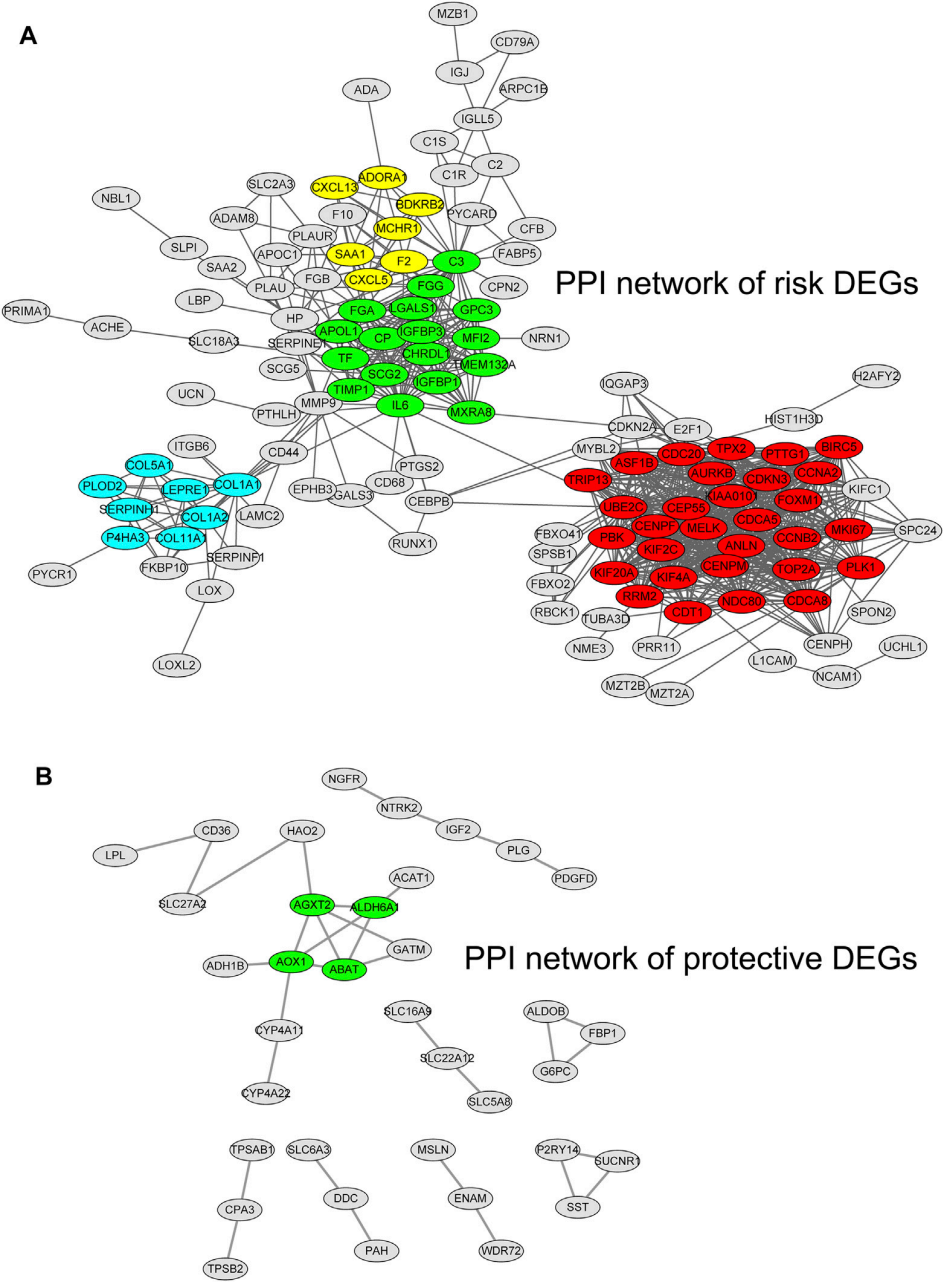
Protein–protein interaction networks for hypoxia–immune-related risk differentially expressed genes (DEGs) and protective DEGs. The densely connected regions of these two networks are labeled with different colors.

By using the STRING database and Cytoscape software, we constructed two PPI networks for hypoxia–immune-related risk DEGs and protective DEGs, respectively. The PPI network of risk DEGs contained 283 nodes and 825 edges (Figure 5A); the PPI of protective DEGs contained 125 nodes and 106 edges (Figure 5B), and an interaction score >0.7 is considered a high-confidence interaction relationship. We also utilized Cytoscape software to find densely connected regions of these two networks and labeled them with different colors, as shown in Figure 5.

### Construction and Validation of the Hypoxia–Immune-Related Prognostic Model

**FIGURE 6:**
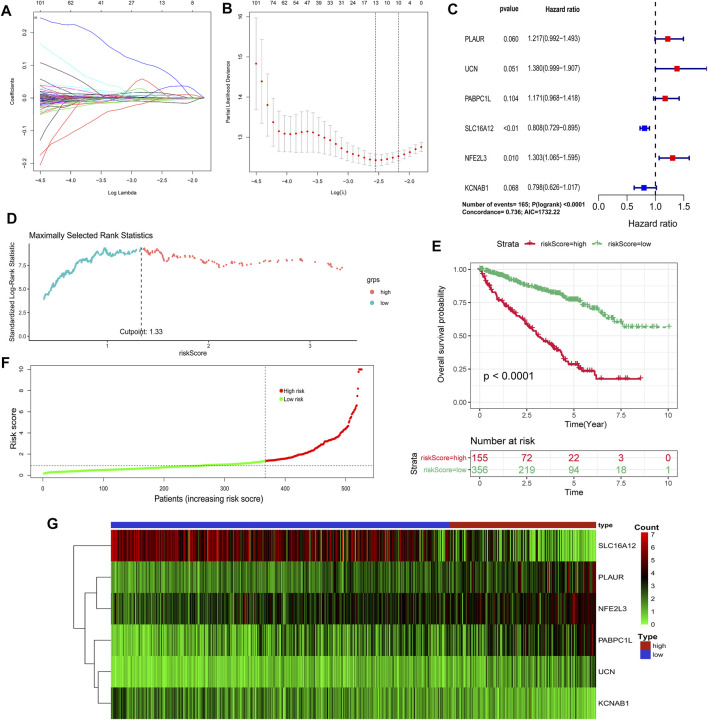
Construction of a hypoxia–immune-related prognostic model. **(A**, **B)** Determination of the number of factors by the LASSO analysis. **(C)** Hazard ratio and *p*-value of genes involved in risk model and some parameters of this risk model. **(D)** Scatter plot of the optimal cutoff value of the risk score. **(E)** Kaplan–Meier plots of overall survival for patients in the risk-high and risk-low groups. **(F)** Hypoxia–immune-related risk score distribution. **(G)** Heat map of the expression profiles of members in the selected 6 genes.

By utilizing univariate Cox regression, multivariable Cox regression, and LASSO regression, we selected a total of six signature genes (PLAUR, UCN, PABPC1L, SLC16A12, NFE2L3, and KCNAB1) from the hypoxia–immune-related gene set to construct a prognostic model, as shown in Figures 6A–C. PLAUR, PABPC1L, SLC16A12, NFE2L3, and KCNAB1 presented significantly different expression levels between tumor tissues and normal tissues (Supplementary Figure S1). Meanwhile, the expression levels of five genes (PLAUR, UCN, PABPC1L, NFE2L3, and KCNAB1) were significantly associated with OS and DFS, and the PABPC1L expression was only related with OS (Supplementary Figures S2, S3). This hypoxia–immune-gene-based model was established to evaluate the survival risk for each TCGA sample as follows: risk score = 0.19639 × (expression of PLAUR) + 0.3222 × (expression of UCN) + 0.15812 × (expression of PABPC1L) − 0.21351 × (expression of SLC16A12) + 0.26479 × (expression of NFE2L3) − 0.22544 × (expression of KCNAB1). The optimal cutoff value of the risk score was 1.33, which was calculated by the “surv_cutpoint” algorithm (Figure 6D). We classified the patients into high-risk and low-risk groups based on this cutoff value. There was a significant prognostic difference between patients from these two groups, which meant that patients with a high-risk score had a higher mortality rate than those with a low-risk score (Figure 6E). It can also be seen from the risk curve and heat map (Figures 6F,G) that these five signature gene expression patterns were different between the high-risk group and low-risk group.

**FIGURE 7:**
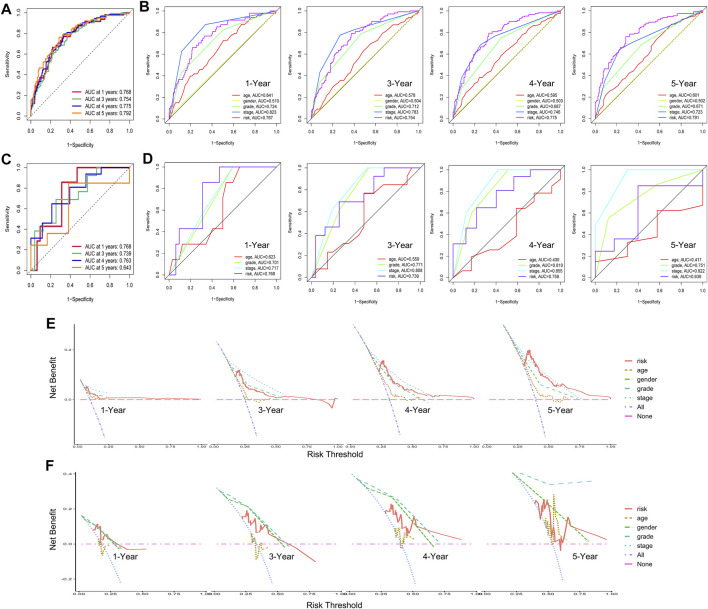
Internal verification and external verification for hypoxia–immune-related prognostic model. **(A)** Receiver operating characteristic (ROC) curve for 1-, 3-, 4-, and 5-year overall survival (OS) in samples of internal verification. **(B)** ROC analysis for other clinical features in samples of internal verification. **(C)** ROC curve for 1-, 3-, 4-, and 5-year OS in external verification. **(D)** ROC analysis for other clinical features in samples of external verification. **(E**, **F)** Decline curve analysis curves for 1-, 3-, 4-, and 5-year OS in samples of internal and external verification.

Then, we conducted a series of analyses to verify the effectiveness and sensitiveness of the prognostic model constructed as detailed above, including internal verification (TCGA samples) and external verification (GSE29609 samples). For the internal verification part, ROC analysis was conducted firstly. The area under the curve (AUC) values of risk score for 1-, 3-, 4-, and 5-year OS were 0.768, 0.754, 0.775, and 0.792, respectively (Figure 7A). Compared with other clinical features, including clinical stage and pathological grade, the ROC analysis again indicated that the risk score was as great as the clinical stage and better than the other clinical features, as shown in Figure 7B. For the external verification part, the AUC values of risk score for 1-, 3-, 4-, and 5-year OS were 0.768, 0.739, 0.763, and 0.643 respectively, which were similar to the results of the internal verification. Moreover, the results of a further ROC analysis to compare with clinical features also had consistency with the internal verification. Moreover, we carried out the DCA analysis for internal and external verification to determine the clinical usefulness of the gene risk model by quantifying the net benefits at different threshold probabilities. As shown in Figures 7E,F, the characteristic of the risk score had excellent clinical effectiveness, which was similar to the clinical stage.

### The Construction of a Nomogram Depends on the Hypoxia–Immune-Related Model and Clinical Features

**FIGURE 8:**
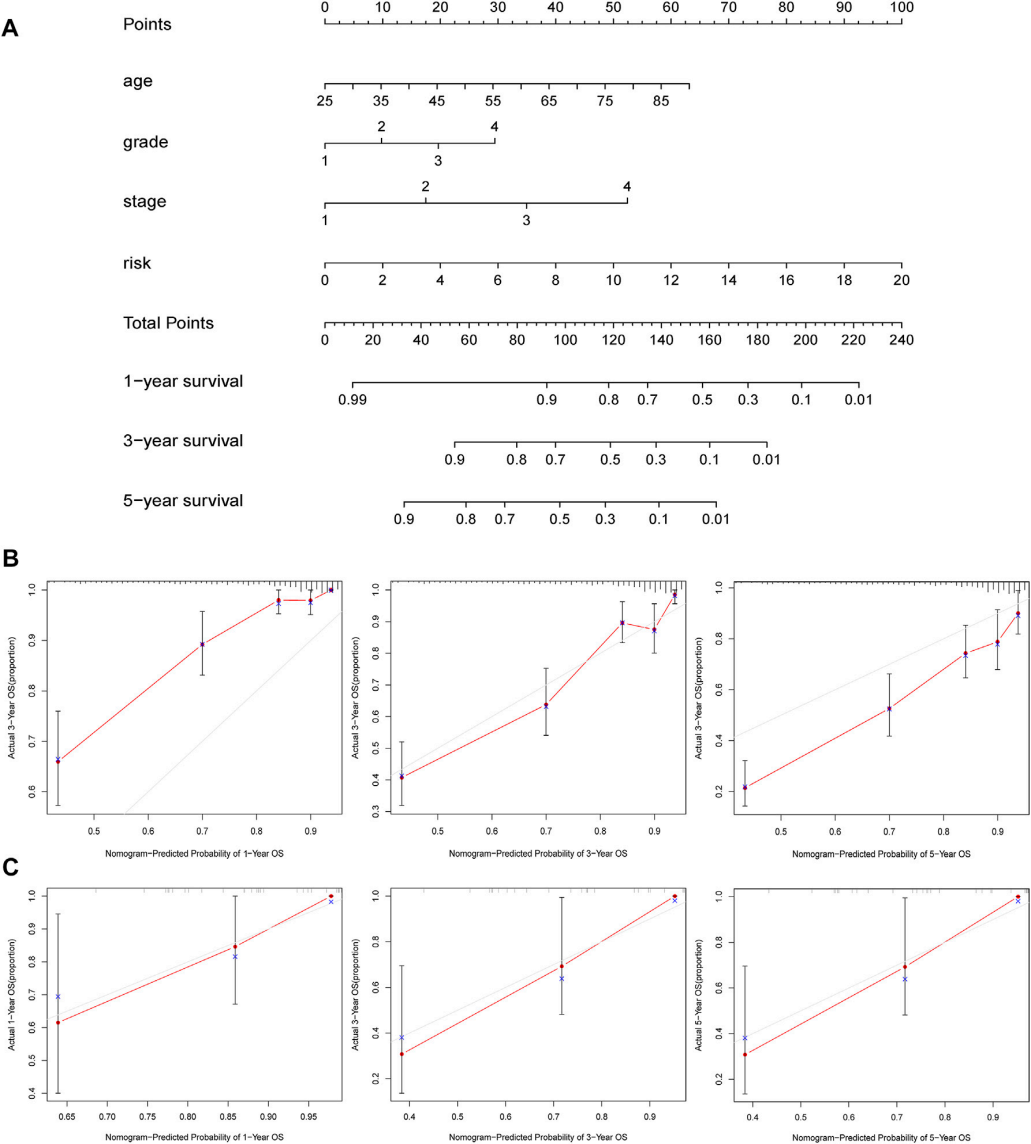
Construction and validation of nomogram. **(A)** Nomogram to predict the 1-, 3-, and 5-years overall survival. **(B**, **C)** Calibration curves for the nomogram model in samples of internal and external verification.

To evaluate the clinical features and the hypoxia–immune-related prognostic model for KIRC prognosis, we integrated the predictive model and some clinical characteristics, including age, clinical grade, and pathological grade, to build a nomogram (Figure 8A). In addition, as shown in Figures 8B,C, we also portrayed the corresponding calibration plots in 1, 3, and 5 years for internal and external verification. Furthermore, it was found that the performance of the nomogram was excellent, especially in predicting 3- and 5-year OS.

### Screening of Small-Molecule Drugs Based on Hypoxia–Immune-Related DEGs

**FIGURE 9:**
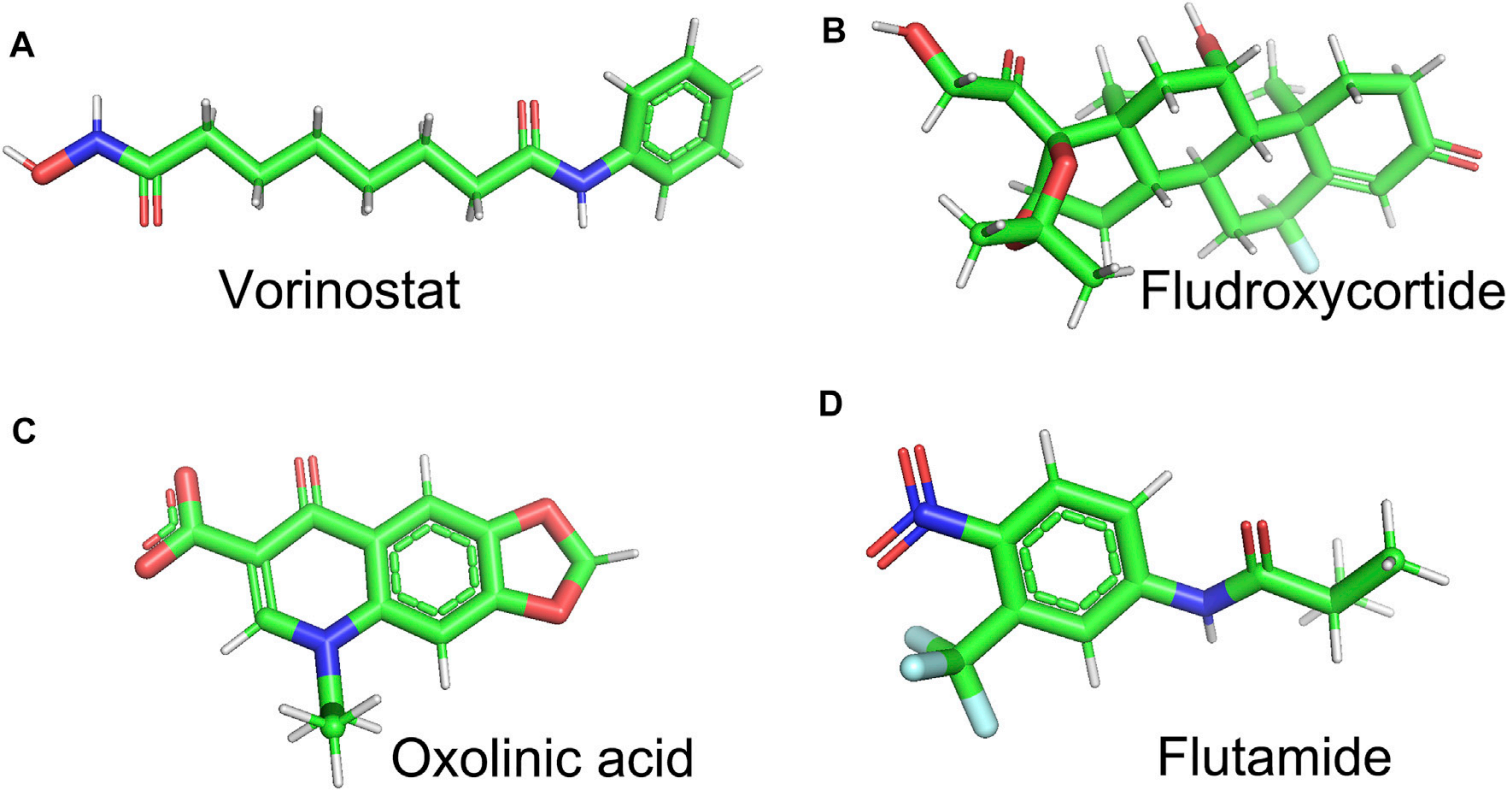
3D structures of vorinostat, fludroxycortide, oxolinic acid, and flutamide represented by Pymol software.

Finally, we employed the CMap dataset to screen some small-molecule drugs, which can potentially treat KIRC patients in the hypoxia–immune-related high-risk group. We predicted these small-molecule drugs with highly significant correlations based on the aforementioned hypoxia–immune-related DEGs. As shown in Table 2, vorinostat, fludroxycortide, oxolinic acid, and flutamide showed a significantly negative association with the high-hypoxia and low-immune status and implied a great possibility in clinical application to alleviate or even reverse the status about severe hypoxia of the tumor microenvironment and low infiltration of immune cells. In addition, the 3D structures of these four molecules are displayed in Figure 9.

**TABLE 2:** Results of the CMap dataset.

CMap name	Mean	*N*	Enrichment	*P*	% non-null
Vorinostat	−0.486	12	−0.614	0	75
Fludroxycortide	−0.5	5	−0.6	0.02906	80
Oxolinic acid	−0.499	5	−0.672	0.00881	80
Flutamide	−0.439	5	−0.598	0.02988	80

## Discussion

KIRC is one of the most common malignancies threatening public health and creating a significant global health burden. It is often diagnosed in the advanced stage due to less notable clinical symptoms in the early stage (Bray et al., 2018). TNM staging is a classical manner to predict the prognosis of KIRC, which is based on clinical information. However, TNM staging does not consider any genetic features. Hence, to provide personalized treatment, it is of great value to identify some genetic features and construct a prognostic model to stratify patients with different risks and prognoses.

We selected the two characteristics of hypoxia and immune to conduct a series of analyses and construct a prognostic model. As the hypoxia in the tumor microenvironment is complicated and varied, it is not valid to determine the hypoxia status by a single biomarker (Liu et al., 2020). Therefore, we utilized the t-SNE algorithm, which is a classical type of machine learning method and provides a robust dimensionality reduction approach. Moreover, t-SNE algorithm has been used in subtype classification in prostate cancer, breast cancer, and gastric cancer (Ahmed et al., 2018; Guo et al., 2019; Liu et al., 2020). As for the immune status, we adopted the ImmuCellAI method to calculate the infiltration of immune cells in KIRC samples. This method supported the ssGSEA enrichment score of expression deviation profile per cell type and has been widely used in cancer research, including pituitary adenomas, hepatocellular carcinoma, sarcoma, and lung adenocarcinoma (Wang et al., 2020a; Zhang et al., 2020b; Hu et al., 2021; Zhao et al., 2021). We successfully clustered the KIRC patients into different groups by hypoxia and immune status using these combined methods. Additionally, our method might be effective and provide a reference for follow-up studies.

By exploring the OS outcomes of patients with different hypoxia and immune status, we found that the high-hypoxia-status samples and low-immune-status samples tend to induce a poor OS outcome, which is consistent with other studies. The hypoxia and immune microenvironment play a critical role in the progression of KIRC (Zhang et al., 2021). Von Hippel Lindau tumor suppressor (pVHL) and HIFs are critical factors in hypoxia-related pathways. With tumor cell proliferation and growth, the hypoxia status of KIRC is more severe and induces the activated HIF signaling pathway to respond to the hypoxia environment (Millet-Boureima et al., 2021). HIF-1 is a master regulator of hypoxia by mainly activating the transcription of some genes to regulate cell angiogenesis, energy metabolism, chromatin remodeling, cell cycle, and even the oxygen-sensing pathway itself, which includes increasing oxygen delivery and facilitating metabolic adaptation to hypoxia (Schödel et al., 2016). The genome-wide analysis showed that more than 500–1,000 genes are associated with the HIF pathway (Mole et al., 2009; Schödel et al., 2011). On the other hand, pVHL is recognized as a component of an E3 ubiquitin–ligase complex, targeting HIFs for proteasomal degradation by tagging them with ubiquitin. However, about 60–80% of KIRC cases display loss-of-function coding mutations in the VHL gene, causing accumulation and stabilization of HIF-α and subsequent transcriptional responses to increase tumor oxygenation (Schödel et al., 2011; Schödel et al., 2016). KIRC is considered to be an immunogenic tumor and has shown high effectiveness for immune therapy, especially immune checkpoint inhibitors and also known to mediate immune dysfunction by stimulating the infiltration of some immune inhibitory cells, such as regulatory T cells (Tregs) and myeloid-derived suppressor cells, into the microenvironment (Vuong et al., 2019; Díaz-Montero et al., 2020). The hypoxia microenvironment can influence the infiltration of immune cells. The immune microenvironment of KIRC appears to be uncommon in some respects, including tumor-infiltrating lymphocytes, Treg, and dendritic cells, which are correlated with disease recurrence and worse survival (Giraldo et al., 2015; Giraldo et al., 2017). A recent transcriptomic and proteomic analysis found that HIF-2α-deficient tumors are related to antigen presentation, CD8^+^ T cell infiltration, and activation, and the single-copy loss of HIF1A or high levels of HIF2A expression is correlated with a higher T cell abundance, all of which indicated that the HIF1 pathway appears to affect T cell inflammation (Hoefflin et al., 2020). However, the mechanisms have not been identified and will require further study.

Our research identified some essential signature genes in KIRC, which are also connected with hypoxia and the immune microenvironment. These genes are PLAUR, UCN, PABPC1L, SLC16A12, NFE2L3, and KCNAB1, and some of them have been previously reported in multiple types of cancer. The plasminogen activator receptor (PLAUR) is a glycosylated, glycan lipid-anchored membrane protein, which has been proven to be a prognostic marker and has a potential role in therapeutic implications (Li and Chen, 2011; Wang et al., 2020b). It binds and activates PLAU, which can convert plasminogen to active plasmin, which can degrade components of the extracellular matrix, thus facilitating invasion and metastasis (Li et al., 2013; K. Lund et al., 2011). Studies also found that PLAUR is related to some vital signaling pathways (as PI3K/Akt and ERK), thus inducing cell migration and proliferation (Aguirre-Ghiso et al., 2003; Nowicki et al., 2011). In addition, recently, research found that PLAUR secretes several cytokines and chemokines and initiates inflammatory responses in macrophages and fibroblast-like synoviocytes through activation of the PI3K/Akt signaling pathways in rheumatoid arthritis (Dinesh and Rasool, 2018). That indicts that PLAUR may also relate to the infiltration of immune cells. Urocortin (UCN) is a 40-amino-acid peptide, which has a prognosis value in KIRC patients, and it is involved in the regulation of angiogenesis and inhibition of proliferation (Tezval et al., 2009; Wan et al., 2019). Poly(A) binding protein, cytoplasmic 1-like (PABPC1L) is an important paralog of PABPC1, regulating and stabilizing the mRNA translation. The depletion of PABPC1L can inhibit the expression level of p-AKT and p-PI3K and suppress the process of proliferation, migration, and invasion in colorectal cancer cells (Wu et al., 2019). Nevertheless, no study has investigated the roles of PABPC1L in KIRC and its relationship with the immune and hypoxia microenvironment. Solute carrier family 16, member 12 (SLC16A12) participates in the transport of creatine, with a high expression level in the normal kidney tissue and a low expression level in KIRC tissue, which indicates that a decreased expression of SLC16A12 is a poor prognostic factor in KIRC (Mei et al., 2019). The nuclear factor erythroid 2-like 3 (NFE2L3) participates in constructing the basic-region leucine zipper family of transcription factors, which is an important factor in tumor progression (Sun et al., 2019). It can promote cell proliferation and metastasis and induce “”EMT of hepatocellular carcinoma *via* activation of the Wnt/β-catenin pathway (Ren et al., 2020). Research also demonstrated that NFE2L3 functions as a critical regulator in a pathway that links NF-κB signaling to control CDK1 activity, thereby driving colon cancer cell proliferation (Bury et al., 2019). As for KCNAB1, most studies showed that the potassium channel, voltage-gated subfamily A regulatory beta subunit 1(KCNAB1) is related to neurologic disorders and diseases. However, few studies focused on the influence on tumor progression (Busolin et al., 2011; Tiong et al., 2019).

We constructed a prognostic risk model based on these six hypoxia–immune genes. The internal verification showed that the 1-, 3-, 4-, and 5-year OS were 0.768, 0.754, 0.775, and 0.792, respectively. The AUC values of the external verification for 1-, 3-, 4-, and 5-year OS were 0.768, 0.739, 0.763, and 0.643, respectively, and compared with clinical stage and pathological grade, the ROC analysis indicated that our prognostic model was as great as the clinical stage and better than the other clinical features. Moreover, compared with other similar research, our hypoxia–immune-related prognostic model had better effectiveness. Zhong *et al*. developed a prognostic model based on nine RNA-binding protein signatures, and the AUC values of this model were 0.71, 0.66, and 0.69, respectively, for 1-, 3-, and 5-year OS (Zhong et al., 2020). Xu *et al*. constructed a prognostic risk model based on PPAR pathway-related genes. The 5-year AUC score was 0.746, and the 10-year AUC score was 0.825, which indicated that this risk model could accurately predict the 5- and 10-year survival rates of KIRC patients. However, the PPAR-related model involved 13 gene signatures, and their research lacked external verification (Xu et al., 2020). In addition, we also carried out a DCA to determine the clinical usefulness of the gene risk model by quantifying the net benefits at different threshold probabilities. The result showed that our risk model had a strong clinical application value. Afterward, based on our prognostic risk model and some clinical features, we further developed a nomogram. The corresponding calibration demonstrated that the performance of the nomogram was great, especially in predicting the 3- and 5-year OS. All of these indicated that we have successfully constructed a hypoxia–immune-related prognostic risk model.

Finally, we screened vorinostat, fludroxycortide, oxolinic acid, and flutamide from many small-molecule drugs. These four drugs showed a significantly negative association with the high-hypoxia and low-immune status, which may alleviate or reverse the status about severe hypoxia of the tumor microenvironment and low infiltration of immune cells, thus improving the prognosis of patients with KIRC.

Among the four drugs mentioned above, vorinostat and flutamide have been used for the treatment of cancer. Vorinostat is a histone deacetylase (HDAC) inhibitor that was approved by the US Food and Drug Administration for the treatment of cutaneous T cell lymphoma. HDAC inhibitors have an effect on anti-angiogenesis by altering the VEGF signaling pathway. A combination of selumetinib and vorinostat can inhibit proliferation and spheroid formation, which is associated with an increase in apoptosis, cell cycle arrest, and reduced cellular migration and VEGF-A secretion in CRC cells (Deroanne et al., 2002; Morelli et al., 2012). Vorinostat also has been recognized as having a correlation with some immune cells in cancer. A study found that it can enhance the sensitivity of cervical cancer cells to the NK cell-mediated cytolytic reaction through the PI3K/Akt pathway (Xia et al., 2020). It also enhances trastuzumab-mediated, antibody-dependent, cell-mediated phagocytosis, which supports a rationale combined treatment approach with trastuzumab for cancer treatment (Laengle et al., 2020). Moreover, vorinostat can down-regulate B7-H1 expression through impairing IFN-γ signaling, which can induce the percentage of tumor-infiltrating CD8+ T cells in gastric cancer (Deng et al., 2018). Flutamide is a nonsteroidal antiandrogen that acts by binding to and blocking intracellular androgen receptors, which is an antineoplastic agent (Brogden and Clissold, 1989; Mariappan and Sundaraganesan, 2014). Research has demonstrated that androgen-mediated suppression of immune reactivity increases the threshold for autoimmunity to develop but likely lowers the threshold for cancer (Gubbels Bupp and Jorgensen, 2018). Meanwhile, studies also found that androgen deprivation therapy (ADT) leads to cell death and infiltration by lymphocytes, and combining ADT with immunotherapy can improve the efficacy of prostate cancer immunotherapy (Gamat and McNeel, 2017). Moreover, the regulation of hypoxia is related to androgen. It has been identified that androgens can regulate VEGF levels through HIF activation in prostate tumors, and inhibition of androgen receptor and HIF may provide a new therapeutic option (Boddy et al., 2005).

## Conclusion

In conclusion, hypoxia and immune status are correlated with the prognosis of patients with KIRC. Some hypoxia–immune-related genes are identified, and a prognostic model is also constructed, which is the first prognostic model based on hypoxia–immune-related signatures and has a better value of effectiveness and clinical application. Finally, a few small-molecule drugs are screened that may alleviate severe hypoxia of tumor microenvironment and low infiltration of immune cells, thus improving the prognosis of KIRC.

## Data Availability

Publicly available datasets were analyzed in this study. This data can be found here: https://portal.gdc.cancer.gov/.
