# Supplementary figures and images for: Identification of Hypoxia–Immune-Related Gene Signatures and Construction of a Prognostic Model in Kidney Renal Clear Cell Carcinoma
Source: Front Cell Dev Biol. 2022 Feb 8;9:796156. doi: 10.3389/fcell.2021.796156 (PMC8860910; doi:10.3389/fcell.2021.796156)

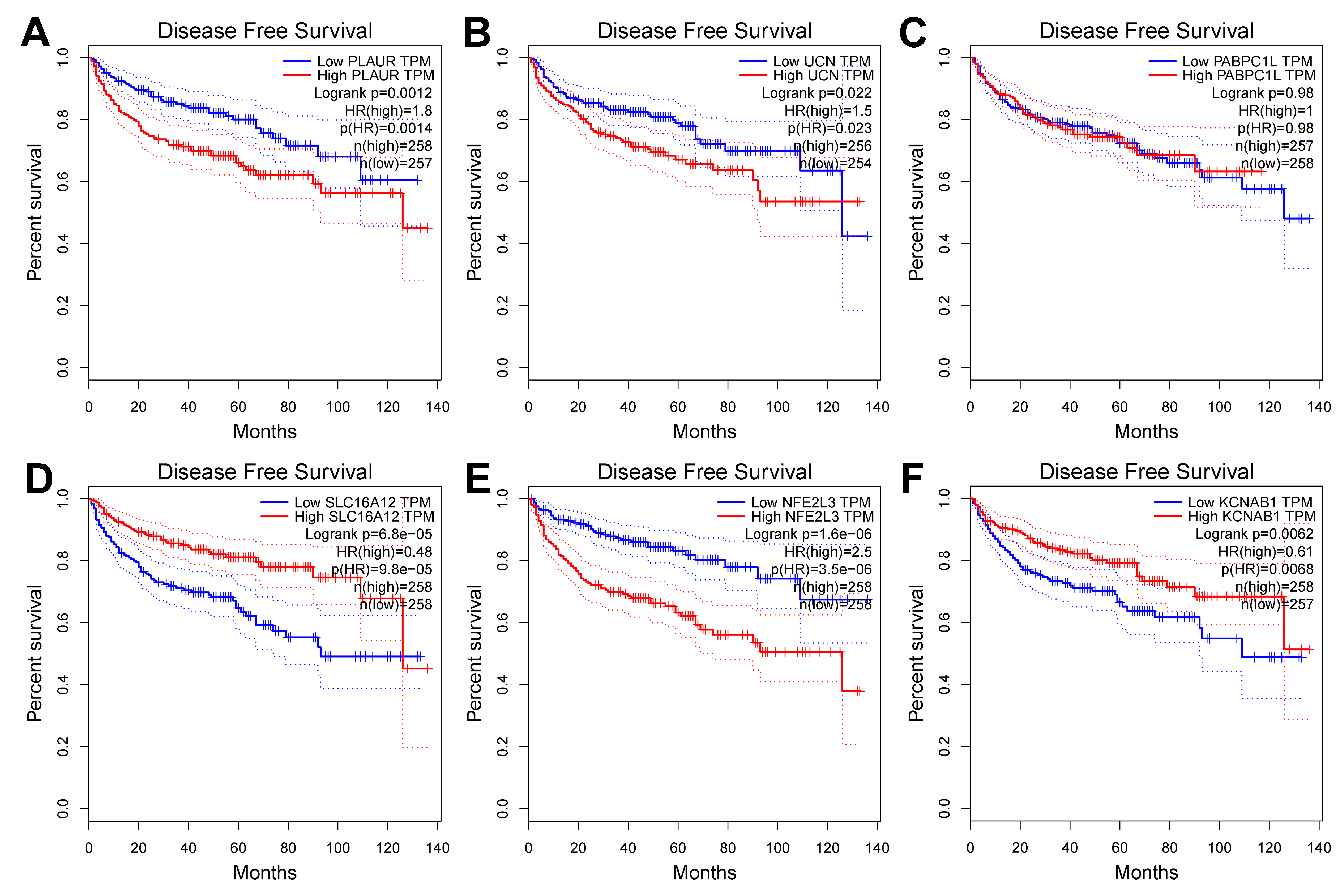

Supplement: Supplementary file 3 [file Image3.TIF]

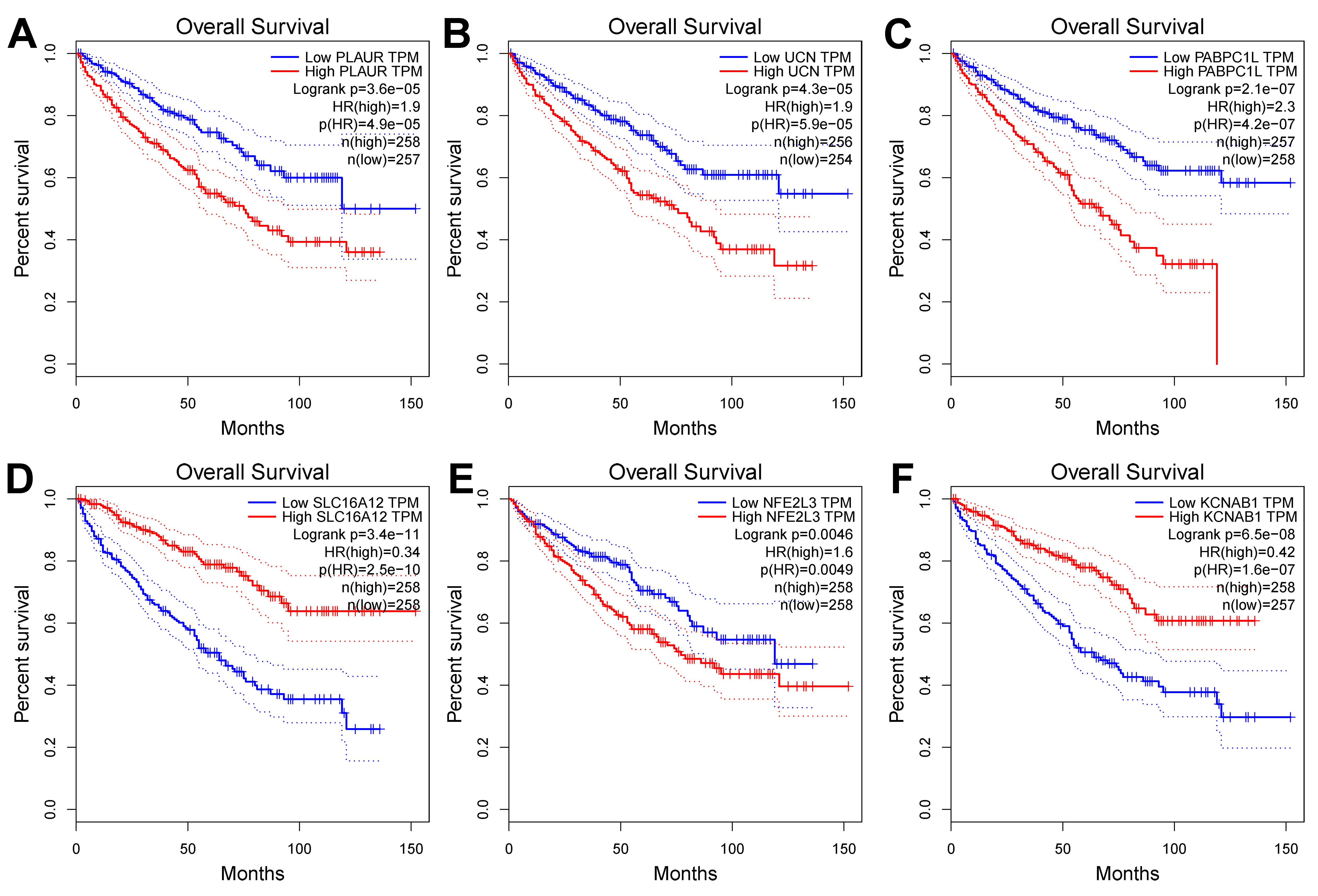

Supplement: Supplementary file 4 [file Image2.TIF]

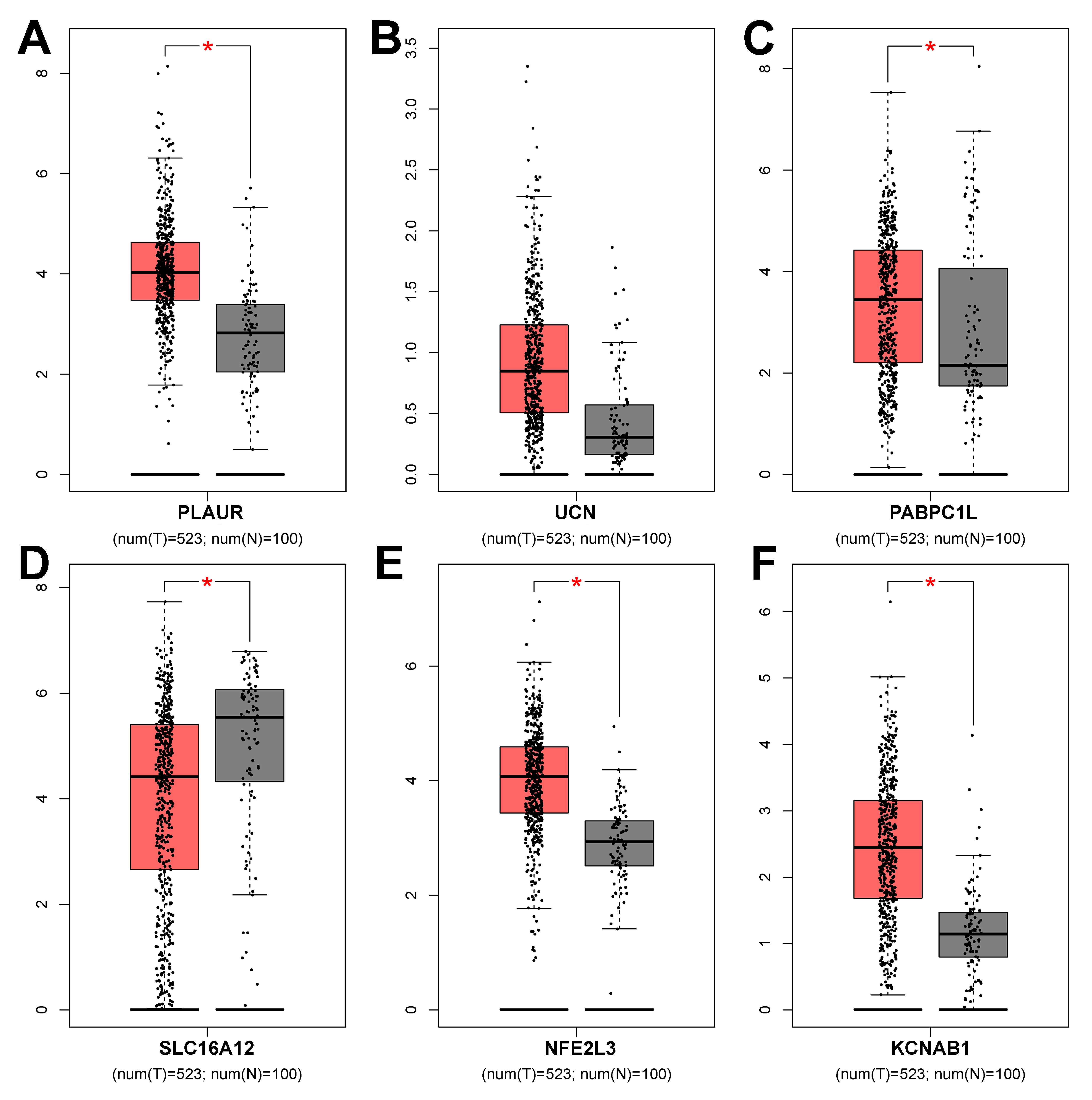

Supplement: Supplementary file 5 [file Image1.TIF]
